# Tolvaptan safety in autosomal-dominant polycystic kidney disease; a focus on idiosyncratic drug-induced liver injury liabilities

**DOI:** 10.1093/toxsci/kfae142

**Published:** 2024-11-04

**Authors:** Sean Hammond, Xiaoli Meng, Jane Barber, Merrie Mosedale, Amy Chadwick, Paul B Watkins, Dean J Naisbitt

**Affiliations:** Department of Pharmacology and Therapeutics, Centre for Drug Safety Science, University of Liverpool, Liverpool, L69 3GE, United Kingdom; ApconiX, Alderley Edge, SK10 4TG, United Kingdom; Department of Pharmacology and Therapeutics, Centre for Drug Safety Science, University of Liverpool, Liverpool, L69 3GE, United Kingdom; ApconiX, Alderley Edge, SK10 4TG, United Kingdom; Division of Pharmacotherapy and Experimental Therapeutics, UNC Eshelman School of Pharmacy, Chapel Hill, NC 27599, United States; Department of Pharmacology and Therapeutics, Centre for Drug Safety Science, University of Liverpool, Liverpool, L69 3GE, United Kingdom; Division of Pharmacotherapy and Experimental Therapeutics, UNC Eshelman School of Pharmacy, Chapel Hill, NC 27599, United States; Department of Pharmacology and Therapeutics, Centre for Drug Safety Science, University of Liverpool, Liverpool, L69 3GE, United Kingdom

**Keywords:** hypersensitivity, drug induced liver injury, tolvaptan, T-cell, idiosyncratic, ADPKD

## Abstract

Tolvaptan is a vasopressin V2 receptor antagonist which has proven to be an effective and mostly well-tolerated agent for the treatment of autosomal-dominant polycystic kidney disease. However, its administration is associated with rare but serious idiosyncratic liver injury, which has warranted a black box warning on the drug labels and frequent monitoring of liver blood tests in the clinic. This review outlines mechanistic investigations that have been conducted to date and constructs a working narrative as an explanation for the idiosyncratic drug-induced liver injury (IDILI) events that have occurred thus far. Potential risk factors which may contribute to individual susceptibility to DILI reactions are addressed, and key areas for future investigative/clinical development are highlighted.

Tolvaptan (Jynarque/Jinarc/Samsca-Otsuka Pharmaceuticals Co.) is a nonpeptide competitive arginine vasopressin receptor 2 antagonist therapeutically originally indicated in the treatment of clinically significant hypervolemic and euvolemic hyponatremia associated with heart failure, cirrhosis, and syndrome of inappropriate antidiuretic hormone (SIADH) (Schrier et al. 2006; Gheorghiade et al. 2007; Konstam et al. 2007; Pose-Reino et al. 2021). No concerns regarding liver safety to tolvaptan emerged during clinical trials for these indications.

More recently, tolvaptan has undergone repositioning for long-term therapeutic use in autosomal-dominant polycystic kidney disease (ADPKD). For ADPKD, tolvaptan has exhibited disease-modifying efficacy. Indeed, reduced progression in total kidney volume and a decreased slope of eGFR decline were reported within key clinical trials including the Tolvaptan Efficacy and Safety in Management of Autosomal Dominant Polycystic Kidney Disease and Its Outcomes (TEMPO) 3:4 (NCT00428948), TEMPO 4:4 (NCT01214421, open-label extension), and subsequent REPRISE (NCT02160145) trials (Torres et al. 2012, 2016, 2017). Unfortunately, an imbalance of hepatic safety signals was revealed upon the un-blinding of TEMPO 3:4. In this study, 40/957 (4.6%) patients receiving tolvaptan versus 5/484 (1%) receiving placebo exhibited serum alanine aminotransferase (ALT) levels exceeding triple the upper limit of normal (ULN) (Watkins et al. 2015). Furthermore, in the open-label extension (TEMPO 4:4), patients previously treated with a placebo were observed to develop a similar incidence of ALT elevations, further supporting liver safety concerns (Watkins et al. 2015). This was recapitulated in REPRISE where ALT elevations >3 x ULN were observed in 38 patients (5.6%) receiving tolvaptan, compared with 8 (1.2%) receiving placebo (Alpers et al. 2023). Most concerning was the observation that 3 patients in TEMPO 3:4 and 4:4 experienced delayed onset, severe liver injury indicated by concomitant elevations in serum bilirubin and ALT (“Hy’s Law cases”) (Temple 2006; Watkins et al. 2015).

Thus, tolvaptan has emerged clinically as an efficacious and important therapeutic in ADPKD with “orphan drug” status, with the caveat of potential for infrequent, but potentially serious drug-induced liver injury. This review outlines the progress made in defining mechanistic aspects of tolvaptan-associated liver injury over the last decade.

##  

### Background on ADPKD

ADPKD is a chronic disease characterized by the progressive formation of renal cysts (Boucher and Sandford 2004). It has been identified as one of the leading genetic causes of end-stage kidney disease (Torres et al. 2007). The prevalence of ADPKD is disputed; with estimates ranging from 1 in 400–4000 (Earle 1958; Iglesias et al. 1983; Davies et al. 1991; Simon et al. 1996; Higashihara et al. 1998; Torres et al. 2007). At least 2 genetic loci have been identified as important determinants; PKD1 (chromosome 16p13.3) and 2 (chromosome 4q21), which encode polycystin 1 (European Polycystic Kidney Disease Consortium 1994) and 2 (Silvia González-Perrett et al. 2001) respectively. Highly heterologous (Rossetti et al. 2012) mutations in these genes are causative in ∼90% of patients, whereas alternate genetic loci have been speculated for the remaining 10%; such as glucosidase II subunit alpha (Rangan et al. 2016). The etiology of ADPKD in individuals expressing such mutations is likely to be similar to direct PKD1/2 mutations, as defective proteins can impair the function of PKD1 or 2 (Porath et al. 2016). These and other mutated genes are therefore postulated to be instrumental to ADPKD, as overviewed in Table 1. Key morbidities associated with ADPKD are listed in Table 2.

**Table 1.** Overview of genes implicated in polycystic kidney disease.

Gene	Chromosomal location	Protein encoded	Transcript length (kb)	Molecular mass (kD)	Number of mutations identified as definitely pathogenic (http://pkdb.mayo.edu)	References
PDK1	16p13.3	Polycystin 1 (membrane receptor)	14.5	462	868 (9)	(Hughes et al. 1995)
PDK2	4q21	Polycystin 2	5.6	110	162 (27)	(Silvia González-Perrett et al. 2001)
GANAB	11q12.3	(Glucosidase IIα subunit)	21.9	107/110 (splice variants)	N/A	(Porath et al. 2016)
LRP5	11q13.2	LDL receptor-related protein 5	136.6	Variable	N/A	(Cnossen et al. 2016)

Both GANAB and LRP5 have been postulated as potential genetic origins in patients/families presenting with ADPKD who lack deleterious PDK1/2 mutations. Reference for database (http://pkdb.mayo.edu) (accessed 10/10/2024).

**Table 2.** Overview of morbidity outcomes associated with ADPKD.

Morbidity	Prevalence in ADPKD population	Reference(s)
Intracranial aneurysm	12.4%	(Xu et al. 2011)
Hepatic cysts	83% overall, strongly correlated to age	(Bae et al. 2006)
End-stage renal disease	Approximately 50% will progress to this stage (predominantly those harboring the PKD1 genotype)	(Dell et al. 2009)
Nephrolithiasis	20%	(Torres et al. 1993)

### Mechanism of action of tolvaptan

Mechanistically, tolvaptan exerts pharmacological efficacy by antagonizing the actions of vasopressin (antidiuretic hormone), via selective (29-fold selectivity for V_2_R over V_1_R) blockade of vasopressin V2 receptors (Garcha and Khanna 2011) in the kidney. Downstream, this reduces the expression and functional insertion of aquaporin proteins. This in turn reduces water reabsorption from the distal convoluted tubule and collecting duct, and results in the aquaretic effect of the drug (Fig. 1). These properties have led to tolvaptan’s use as a masking agent; hence the prohibition by the world antidoping agency and incorporation into doping screening (Rzeppa and Viet 2016). Additionally, disease-modifying effects of tolvaptan in ADPKD have been postulated (Reif et al. 2011), with the proposed mechanism shown below (Fig. 2).

**Fig. 1.**
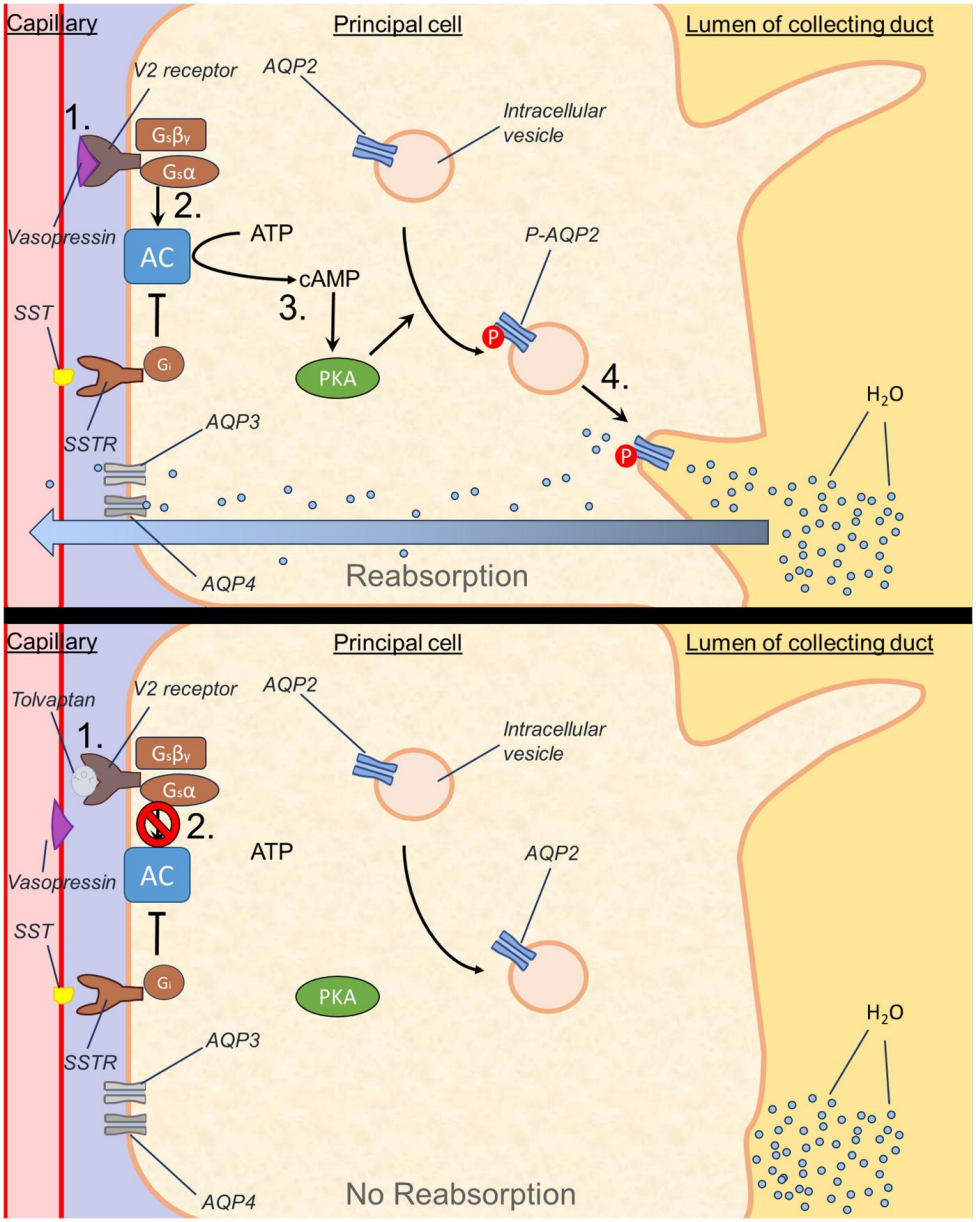
Aquaretic mechanism of action of tolvaptan (TOL). (**Upper panel**) Physiological action of vasopressin: Vasopressin (AVP) binds V2R receptors (**1**), activating G2 proteins (**2**), which in turn activate adenylyl cyclase (AC). AC cleaves ATP; leading to an increase in intracellular cAMP, which drives activation of protein kinase A (PKA) (**3**) and subsequent aquaporin 2 channel expression and insertion into the lumen of the collecting duct (**4**). The net result is that water is reabsorbed. (**Lower panel**) Pharmacological action of tolvaptan: Tolvaptan competitively inhibits the action of vasopressin at V2R receptors (**1**), resulting in the blockade of the steps described above (**2**). Abbreviations: AC, adenylyl cyclase; SST, somatostatin; SSTR, somatostatin receptors; cAMP, cyclic adenosine monophosphate; PKA, protein kinase A; AQP2, aquaporin 2; P-AGP2, phosphorylated aquaporin 2.

**Fig. 2.**
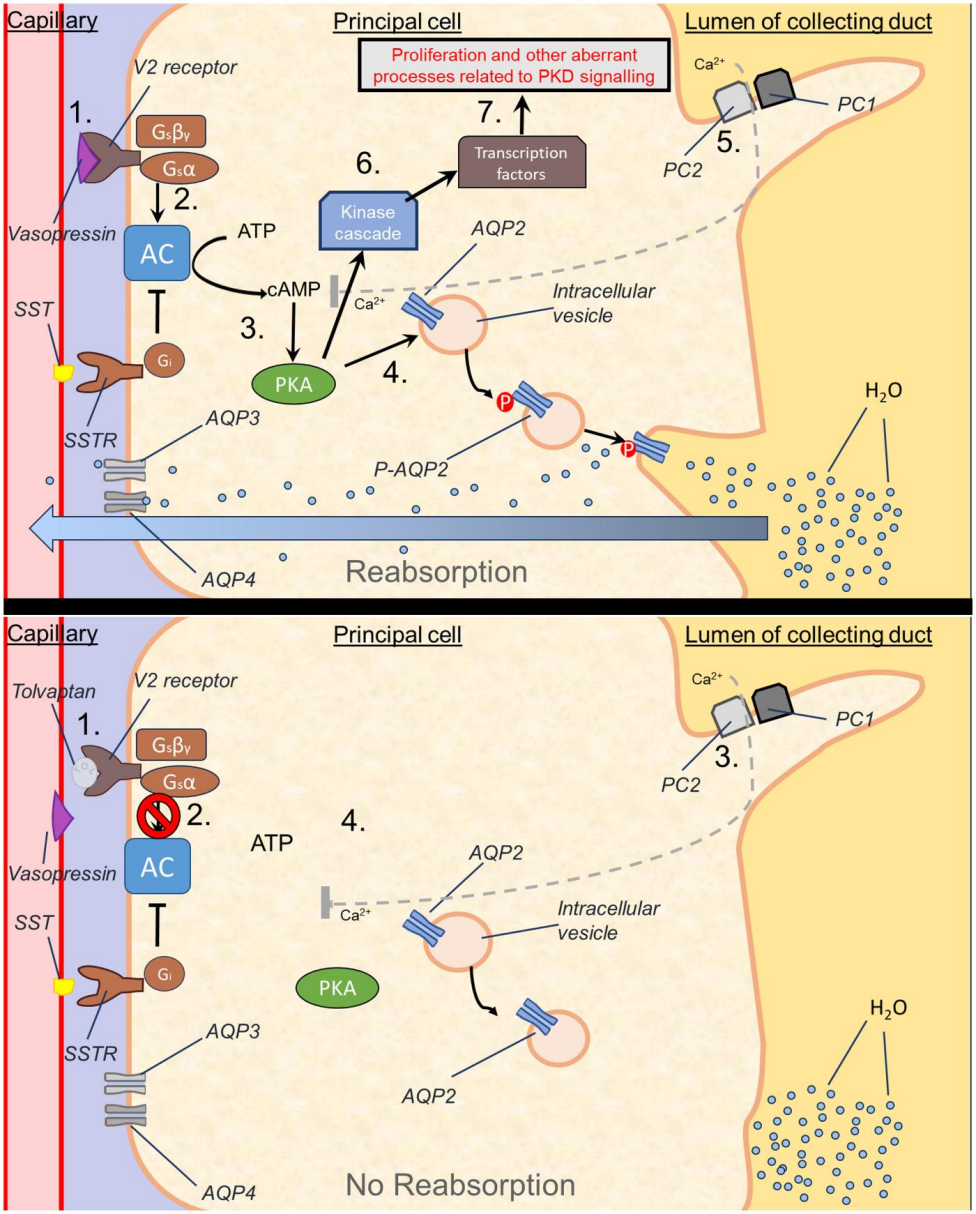
Proposed mechanism of action by which tolvaptan exerts disease-modifying effects in ADPKD. (**Upper panel**) Simplified overview of disease processes involved in ADPKD: Vasopressin (AVP) binds V2R receptors (**1**), activating G2 proteins (**2**), which in turn activate adenylyl cyclase (AC). AC cleaves ATP, thereby increasing intracellular cAMP, which leads to activation of protein kinase A (PKA) (**3**). This drives the water reabsorption pathway (**4**) as outlined in Fig. 1, but also contributes (alongside EGF/VEGF/IGF signaling) to the initiation of a kinase cascade (such as src, Ras, B-raf, MEK, and ERK) and the activation of transcription factors which lead to various aberrant disease-related features (abnormal cellular metabolism, cellular proliferation, impaired tubulogenesis, etc.) (**6–7**). Proposed theoretical role of ADPKD in pathway; The intake of intracellular calcium via the PC1/PC2 mediated pathway serves to inhibit adenylyl cyclase activity (possibly AC6) and thus inhibits the pathway outlined above. In the setting of ADPKD, PC1/PC2 mutants result in defective calcium shuttling by these proteins, leading to reduced intracellular calcium, oscillations in intracellular calcium concentration in ADPKD patients therefore do not adequately provide negative feedback to the cAMP: PKA pathway (**5**). (**Lower panel**) Tolvaptan competitively inhibits the action of vasopressin at V2R receptors (**1**), resulting in the blockade of steps described above (**2**), reducing the overall activation status of the cAMP pathway despite the (theoretical) ADPKD-related changes to intracellular calcium signaling (**3**), leading to reduced activity of the pathological pathways (**4**). Abbreviations: AC, adenylyl cyclase; SST, somatostatin; SSTR, somatostatin receptors; cAMP, cyclic adenosine monophosphate; PKA, protein kinase A; AQP2, aquaporin 2; P-AGP2, phosphorylated aquaporin 2; PC1, polycystin 1; PC2, polycystin 2. Figure adapted and simplified from multiple sources (Reif et al. 2011; Chebib et al. 2015; Capuano et al. 2022; Bakaj and Pocai 2023; Zhou and Torres 2023).

Pharmacokinetic parameters of tolvaptan have been evaluated in a number of studies. Approximately 20 phase 1 metabolites have been postulated to exist in vivo (Mazzarino et al. 2017), with two key metabolites and proposed routes of derivation depicted in Fig. 3. Nonclinical evaluation of the pharmacokinetic profile of tolvaptan in rats is published (Furukawa et al. 2011, 2014). Significant sexual dimorphism in the metabolic profile of tolvaptan between male and female rats has been documented: DM-4104, DM-4107, DM-4110, DM-4111, DM-4119, DM-4121, and MOP-21826 all exhibited greater Cmax and AUCt in female rats, whereas DM-4103 was formed in greater quantities in males (Furukawa et al. 2014). Quantitative tissue distribution assessment following single- and repeat-dose tolvaptan administration showed broad tissue distribution with hepatic tissue distribution exceeding that of blood/plasma by multi-fold (>10-fold) margins at early timepoints (Furukawa et al. 2011).

**Fig. 3.**
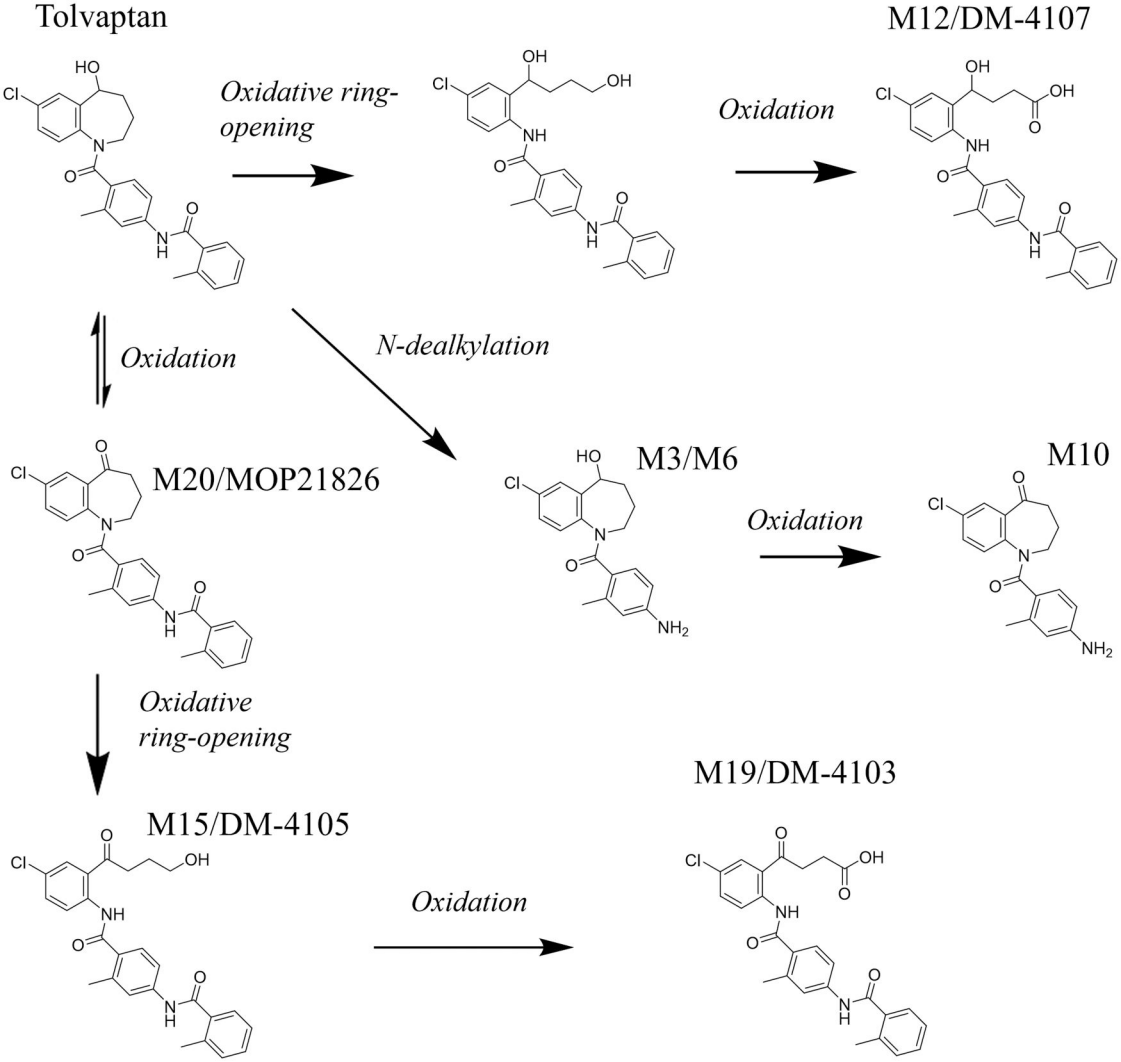
Overview of known tolvaptan major metabolites (DM-4103 and DM-4107) and postulated routes of metabolic derivation. Adapted and simplified from Furukawa et al. (2014) and Mazzarino et al. (2017), *Full metabolic pathways are not depicted, but are available at source publications.

The majority of phase 1 biotransformation of tolvaptan is attributable to cytochrome P450 enzymes; specifically CYP3A4 and CYP3A5 (Bhatt et al. 2014; Shoaf et al. 2017). Since these enzymes exhibit considerable phenotypic variability (due to environmental and genetic influence), the metabolic profile of tolvaptan is subject to inter-patient variability (Mazzarino et al. 2017) and is sensitive to CYP3A inhibitors/inducers (Shoaf et al. 2012). Tolvaptan is also a substrate of P-glycoprotein, as indicated by interactions with digoxin (Shoaf et al. 2011). It is also highly protein bound; ∼98% (Furukawa et al. 2011), and has an absolute mean bioavailability of 56% (Blair and Keating 2015).

### Clinical pattern and ramifications of liver safety signals

The pattern of DILI within the TEMPO trials was assessed extensively in Watkins et al. (2015). A hepatic adjudication committee concluded that in 17 patients presenting with liver injury on the TEMPO 3:4 and 4:4 clinical trials, a causal role of tolvaptan in the liver injury was “probable” or “highly likely.” Three of these patients met Hy’s law criteria; with serum ALT >3 times the ULN, and concomitant bilirubin elevations >2-fold ULN. Observations included a general latency of onset to hepatic injury of 3–18 months from initiation of the drug, a high incidence (∼75%) of hepatic cysts in affected individuals, and the indication of female predominance, with female subjects constituting 60% of subjects in the “probable” or “higher” categories, and accounting for all 3 Hy’s law cases. Twenty-one patients exhibiting ALT elevations were rechallenged with tolvaptan following normalization of ALT, in 10 of these individuals the rechallenge was eventless, permitting the continuation of therapy. Contrastingly, 11 individuals exhibited rapid ALT elevations (Watkins et al. 2015) exemplified by the longitudinal liver function tests for one patient outlined in Fig. 4. This represents a positive rechallenge rate comparable to that exhibited by well-documented immuno-allergic DILI-causing compounds such as halothane (Hunt et al. 2017).

**Fig. 4.**
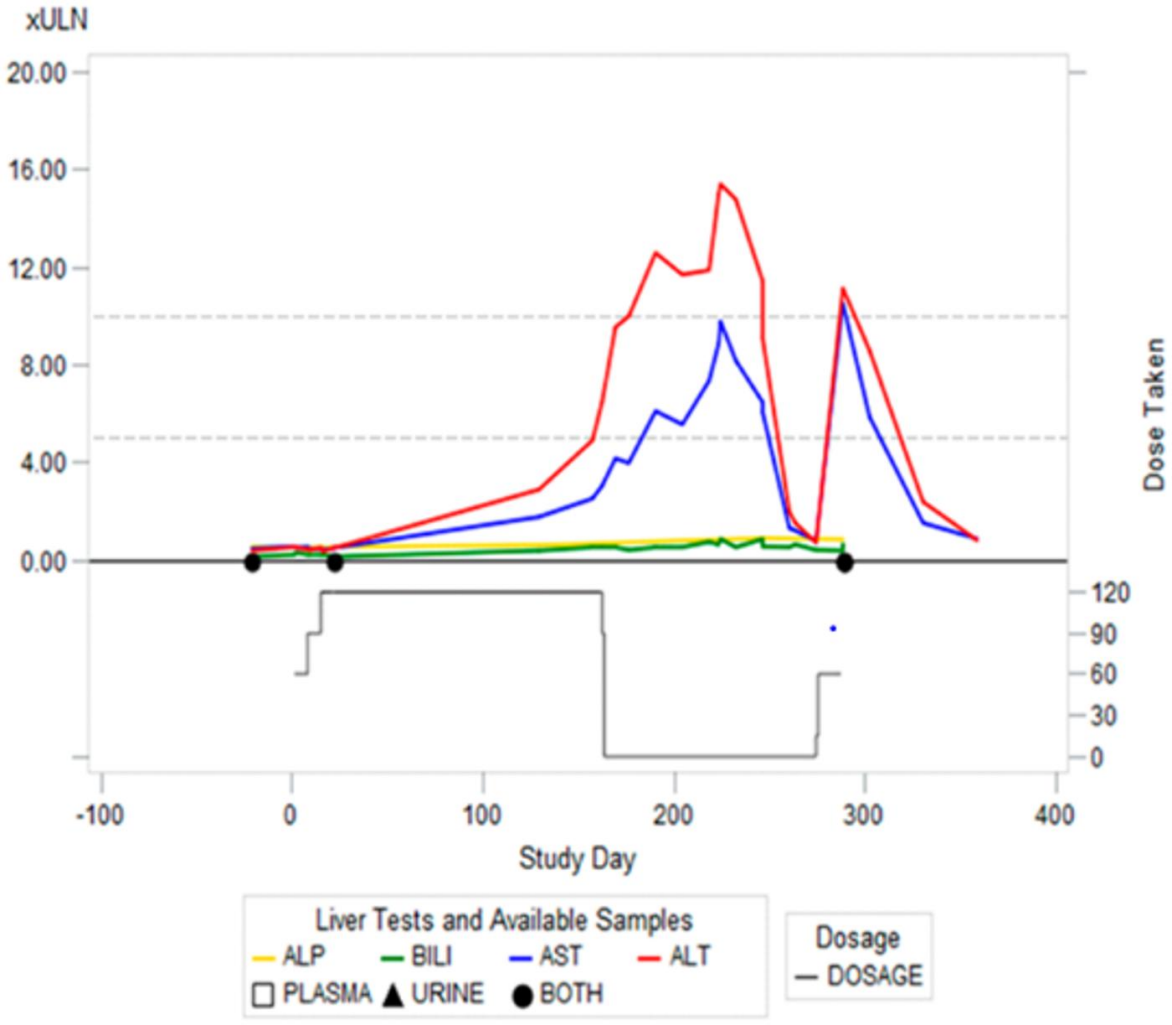
Clinical chemistry profile of a patient who experienced primary liver injury followed by a “positive” rechallenge. Liver function over the study period for a trial subject that was rechallenged at a lower dose. Alkaline phosphatase (ALP), ALT, aspartate aminotransferase (AST), and total bilirubin (BILI) are indicated by traces in the upper compartment. The dose regimen is depicted in the lower compartment. Reproduced with permission from Gibson et al. (2020).

Precautionary measures taken to mitigate this risk include limitation of tolvaptan treatment duration to 30 days in non-ADPKD indications, and avoidance of use in patients with underlying liver disease or cirrhosis (Nursing 2013). Ironically, hyponatremia in patients with cirrhosis is relevant for earlier indications of tolvaptan, and patients with cirrhosis accounted for a large proportion (>25%) of the SALT trial cohorts with no obvious liver safety concerns identified (Torres et al. 2012).

Recently, the systematic evaluation of hepatotoxicity within clinical trials was revisited in subsequent placebo-controlled clinical trials with tolvaptan in ADPKD (Alpers et al. 2023). Once again, an imbalance of hepatic safety signals was observed, and the general profile of DILI was similar with a 2 to 3 month typical time to onset of liver injury following initiation of the drug. In individuals who experienced liver injury, tolvaptan re-exposure resulted in positive rechallenge in the majority (30/38 or 79%) of cases (Alpers et al. 2023). Importantly, no severe toxicities (i.e. acute liver failure or additional “Hy’s Law” cases) occurred in the trials evaluated in these analyses, which supports the intensive liver chemistry monitoring stipulated in the FDA label for JYNARQUE (monthly testing for ALT, AST, ALP, and bilirubin) (FDA 2020). It is worth noting however, that case reports have sporadically appeared in the literature (postmarketing) that detail both mild and severe instances of liver injury with tolvaptan (Makabe et al. 2018; Endo et al. 2019; Pellegrino et al. 2019; Merino Bueno et al. 2022; Alpers et al. 2023). The rate and pattern of tolvaptan-associated liver injury have been shown to be consistent within several postmarketing surveillance studies (Mochizuki et al. 2021; Estilo et al. 2022).

## Mechanistic lines of investigation into tolvaptan-associated drug-induced liver injury

The observation of IDILI associated with tolvaptan is a challenging scenario; tolvaptan progressed through clinical trials and was approved for clinically significant hypervolemic and euvolemic hyponatremia associated with SIADH secretion, heart failure, and cirrhosis, all without significant incidence of such liabilities. Indeed, it was only upon its repositioning (at the stage of phase 3 clinical trials) as a disease-modifying therapeutic option for ADPKD that significant liver safety concerns were raised. As a result, a basic investigation of this liver injury has been pursued in order to: (i) shed light on mechanistic aspects of disease pathogenesis, (ii) delineate how such liabilities were not detected within preclinical studies, and (iii) identify any risk factors associated with reactions.

### Nonclinical toxicology studies

Nonclinical toxicology studies were conducted for tolvaptan in rats and dogs in support of initial human trials of tolvaptan (Table 3). Oral doses up to 1000 mg/kg/day in 26-wk rat and 52-wk dog chronic toxicology studies did not indicate liver injury potential in GLP toxicology studies, though centrilobular hypertrophy was observed at the highest (1000 mg/kg/day) dose group (FDA). Additional, specialized/investigation studies included an investigational study utilizing collaborative cross mice which similarly did not yield overt liver toxicity (Mosedale et al. 2017b) (Table 3) and the following:

**Table 3.** Overview of assorted relevant, in vivo toxicology studies as outlined in regulatory pharmacology reviews for tolvaptan marketed in the form of Jynarque (FDA 2017a), Samsca (FDA 2007) (FDA), and Samsca (EMA 2009), Jinarc (EMA 2015) (EMA).

Study type	Species	Dose level (RoA)	Max safety margin: BSA based conversion	Liver-related safety findings
Single dose	Rat	2000 mg/kg (oral)	162	No overt toxicity findings
Single dose	Dog	2000 mg/kg (oral)	540	No overt toxicity findings
26-wk chronic dosing	Rat	30, 100, 1000 mg/kg daily (oral)	81	No overt toxicity findings Possible increase in enlarged liver finding (though in <15% of study animals—middle and high dose)
52-wk chronic dosing	Dog (beagle)	30, 100, 1000 mg/kg daily (oral)	270	No overt toxicity findings
6-wk repeat dose juvenile	Rat (Sprague–Dawley)	30, 100, 1000 mg/kg daily (oral)	Up to 81-fold	Centrilobular hypertrophy of hepatocytes (reversible)+increased liver weight (high dose only) Significant elevations in total bilirubin
9-wk repeat dose juvenile (4 day old)	Rat (Sprague–Dawley)	10, 30, and 100 mg/kg daily (oral)	–	No overt toxicity findings
Single dose (investigative toxicology)	Mouse (45 strains of collaborative cross)	100 mg/kg (oral)	Approximately 4-fold	No overt liver toxicity ALT, AST, total bilirubin (TBIL), and miR-122 elevations in strain-specific fashion Identification of transcriptomic changes in susceptible strains (discussed further below.

- GLP guinea pig antigenicity tests conducted at doses up to 10 mg/kg SC/IM followed by IV administration: No evidence of systemic active or passive anaphylaxis was observed (FDA 2007).
- Single-dose toxicity of DM-4103 and DM-4107 administered SC (both at 100 and 500 mg/kg doses) in rats: No overt toxicity was detected (FDA 2007).

### Direct toxicological mechanisms

Tolvaptan was investigated within classical HepG2 toxicity assays in vitro, it was found to inhibit cell growth and to cause cell death in a time- and concentration-dependent fashion. The parent drug was found to elicit overt reductions in cellular viability at concentrations below 100×*C*_max_ (3.125–100 µM) (Wu et al. 2015). From these acute studies, key direct toxicological mechanisms within HepG2 cells were found to include delayed cell cycle progression (>20 µM), ROS generation and subsequent DNA damage (>60 µM), and apoptotic induction of cells. A mitochondria-mediated pathway for apoptosis was indicated by reductions in mitochondrial membrane potential and cytochrome C release. Notably, HepG2 cells which overexpressed CYP3A4 (via lentiviral transfection) were not significantly sensitized to tolvaptan cytotoxicity, indicating that the parent drug was primarily responsible for the observed cytotoxicity (Wu et al. 2015). Further in vitro studies from the same group evaluated the role of other metabolic pathways in tolvaptan-induced cytotoxicity, namely sulfation. Overexpression of sulfotransferases induced modest leftward shifts in tolvaptan cytotoxicity curves in HEK293 cell lines (with or without concomitant CYP3A4 overexpression) in vitro (Fang et al. 2016). Additional studies have evaluated tolvaptan and metabolite influence on hepatocyte mitochondrial respiration in vitro. Mitochondrial stress tests *(*seahorse XF analyzer) in hepG2 cells conducted over a dose range of compounds (tolvaptan 0.01–50 μM, DM-4103 0.01–200 μM, DM-4107 0.01–200 μM), showed that tolvaptan and DM-4103 inhibited basal respiratory function (OCR) at the highest concentrations used (Woodhead et al. 2017).

In vitro evaluation within more advanced cell culture platforms mostly reflects these simple in vitro findings. Tolvaptan exposure in primary human hepatocyte coculture models results in hepatocellular stress in a time- and concentration-dependent manner. At concentrations which were not overtly cytotoxic (0.01–50 µM), high-content imaging of cell cultures revealed mitochondrial dysfunction (hepatocytes with both high cytoplasmic cytochrome c spot intensity and low mitochondrial membrane potential) and apoptotic behavior, whereas microarray profiling highlighted the upregulation of oxidative stress and xenobiotic metabolism pathways (Mosedale et al. 2017a). A further key point from these studies was the identification that the content of exosomes released from hepatocytes was altered in these mildly toxic conditions. Specifically, the release of miR-122, a hepatocyte-specific danger signal microRNA, was described (Mosedale et al. 2017a). Spheroid cultures of primary mouse hepatocytes permit longer term exposures to drug and have been studied in the context of direct cytotoxicity of tolvaptan, supporting the idea that longer exposures accentuate observed direct toxicity (Nautiyal et al. 2021).

An important consideration throughout these in vitro experimental studies (and in other investigations outlined below) is the relevance of the in vitro concentrations of drug (metabolites) used relative to an in vivo situation. The mean plasma *C*_max_ of tolvaptan and DM-4107 in plasma of patients repeat-dosed with tolvaptan (90/30 mg) are 1–2 µM, whereas DM-4103 is known to be an accumulating metabolite with a long half-life and reach concentrations >10 µM (Boertien et al. 2013; FDA 2017b; Shoaf et al. 2017). Concentrations utilized within in vitro studies outlined in this manuscript frequently exceed this. However, the obscurity of human hepatic exposure to drugs is a classic challenge with respect to the application of in vitro investigative and predictive DILI models. Indeed, to account for exposure modifying parameters applicable to hepatocytes (first-pass exposure, intraindividual PK variability, accumulation, and diet/drug–drug interactions), it has been proposed that concentrations as high as 100× plasma *C*_max_ can be scientifically justified (Atienzar et al. 2016). With specific reference to tolvaptan, liver concentrations achieved in humans are not well-defined. However, hepatocyte accumulation (∼10-fold incubation concentrations) has been reported within in vitro assays using sandwich-cultured hepatocytes (Lu et al. 2016). Hepatic distribution in vivo has also been illustrated in “The role of ADPKD and other risk factors influencing susceptibility” section; rat tissue distribution studies demonstrated hepatic distribution exceeding that of plasma by at least an order of magnitude at various timepoints in quantitative tissue distribution studies (Furukawa et al. 2011). Overall, concentrations in the liver are likely to be multi-fold higher than the plasma *C*_max_, which justifies the lower concentrations used within many investigative studies. It is likely that the upper bounds of concentrations used within in vitro studies exceed that of typical hepatic exposure. However, given factors described above which introduce considerable intraindividual variability, and disease-related factors (discussed in “The role of ADPKD and other risk factors influencing susceptibility” section), it is possible that the higher concentrations used are still relevant.

### The role of metabolism in tolvaptan toxicity

Tolvaptan is metabolized primarily by 3 major biotransformation pathways: hydroxylation, dehydrogenation, and deamidation, generating more than 20 metabolites in humans. Overall, tolvaptan is principally metabolized by CYP3A4 (Mazzarino et al. 2017), a member of the CYP3A enzyme family (Dai et al. 2001), and so is likely to be subject to a degree of intraindividual variability, as well as pharmacokinetic drug–drug interactions (Shoaf and Mallikaarjun 2012; Shoaf et al. 2011, 2012). Tolvaptan’s complex metabolism (Mazzarino et al. 2017), raises the possibility of reactive metabolite generation and direct protein binding as toxicological mechanisms, with possible Schiff base formation via aldehyde intermediates involved in the formation of azepine-cleaved derivatives. Additionally, deamidation results in an exposed aromatic amine, a moiety that often can be bio-activated to protein-reactive hydroxylamine and nitroso derivatives known to be involved in the toxicity of a plethora of compounds (Castrejon et al. 2010; Alzahrani et al. 2017; Hammond et al. 2022a).

To date, data supporting the existence of (and a toxic role for) reactive metabolites within the context of tolvaptan-associated DILI are scant. Relevant observations in discovery and development include limited CYP inactivation in vitro (an indicator of potential reactivity) (FDA 2007; Orr et al. 2012) and high excretory recovery of tolvaptan in mass balance experiments (∼98.9%) (FDA 2007). Further to this, in subsequent investigative work, the observation of decreased cytotoxicity both in CYP3A4 transduced cell lines (Wu et al. 2015), and in the more metabolically competent hepaRG cell lines (unpublished data) indicate that CYP-catalyzed reactions of tolvaptan do not generate sufficient levels of reactive metabolites to elicit cytotoxicity in vitro. Unfortunately, there is a paucity of published studies directly assessing the capacity for bioactivation of tolvaptan to reactive metabolites (and downstream covalent binding). As such, the potential role of reactive metabolite generation in tolvaptan-associated liver injury cannot be discounted and should be of interest for further investigations. The remainder of the toxicological literature has focused predominantly on the parent drug, and its 2 major, relatively stable metabolites; DM-4103 and DM-4107. Several mechanisms outlined below cover the plausible toxicological mechanism applicable to these compounds.

### Bile acid accumulation

Hepatocyte bile acid transporter inhibition has been proposed as a mechanism of tolvaptan-induced liver injury. Disruption of bile acid homeostasis has been suggested to cause DILI via direct; oxidative stress and mitochondrial dysfunction, as well as indirect; abnormal mitochondrial cristae, cellular membrane disruption (Billington et al. 1980), generation of reactive oxygen species (Sokol et al. 1993), mechanisms. The capacity for tolvaptan and its metabolites to impact upon the activity of bile acid transporters has been investigated in several studies. Slizgi et al. (2016) characterized the inhibitory potential of tolvaptan and its 2 major metabolites on hepatic transporters within sandwich-cultured human hepatocyte cultures in vitro (Table 3). DM-4103 was identified as a clinically relevant inhibitor of NTCP, BSEP, and potentially also MRP4 at the 90-mg therapeutic dose used in the indication of ADPKD (Slizgi et al. 2016). When model bile acids were incorporated into the cultures, tolvaptan was found to promote intracellular accumulation of CDCA, TCDCA, and GCDCA. Other key findings from this study were that tolvaptan accumulated in hepatocytes (to levels as high as 500 µM), and that metabolism of tolvaptan occurred in the primary hepatocytes, with ∼30% of the tolvaptan dose metabolized in 10 minutes (Lu et al. 2016; Slizgi et al. 2016).

With further study, it has become apparent that the indication itself plays an important role in determining the susceptibility of subjects to this mode of toxicity. A relevant feature of the rodent model of ADPKD (the polycystic [PCK] rat) is that they present with greater baseline accumulation of bile acids in the liver, kidneys, and peripheral blood (which may have a mechanistic role in the derivation of cystic cholangiocytes) (Munoz-Garrido et al. 2015). Alterations in hepatic transporter expression, such as an observed 3-fold reduction in MRP2 expression may drive this intrinsic phenotype in the PCK rat (Bezencon et al. 2019). Targeted studies in these animals have been performed with the aim of gaining translational understanding. Isolated perfused livers of PCK rats exhibited greater accumulation of tolvaptan, DM-4103, and DM-4107, and there was significantly greater recovery of DM-4107 in outflow perfusate, as well as significantly reduced biliary excretion of DM-4103 (relative to wild type livers). Taken together and applied to a simulation model, these findings indicate that the impact of ADPKD on the hepatobiliary disposition of tolvaptan is to enhance exposure to the metabolites (Beaudoin et al. 2019). The inhibition of BSEP by tolvaptan (metabolites), and disease-related reductions in MRP2 expression may therefore synergistically attenuate the biliary efflux of bile acids. Additionally, components of adaptive responses that ameliorate hepatocellular overloading with bile acids in cholestasis e.g. MRP4, that provide a route of sinusoidal efflux (Sticova et al. 2018), may also be inhibited as outlined in Table 4. Since DM-4103 has a low IC50 for inhibition of BSEP and MRP4, a role for this metabolite in tolvaptan-associated DILI through hepatocellular bile acid accumulation is plausible. Disease interaction through ADPKD-related hepatic transporter abnormalities (i.e. downregulation of MRP2) may confer susceptibility within the ADPKD population. Furthermore, the accumulating nature of DM-4103 and/or disease progression may also offer an explanation for the latency of DILI onset seen in most cases. A plausible proposition is that once disease progression confers a susceptible state in individuals (e.g. through reduction of MRP2 expression as outlined above; Beaudoin et al. 2021), toxicity may proceed, which could account for the long and variable latency to onset of initial liver injury.

**Table 4.** Transporter inhibition values for tolvaptan, DM-4103, and DM-4107 (Slizgi et al. 2016).

	Inhibitory concentration (IC50) (μM)		
	Tolvaptan	DM-4103	DM-4107
*BSEP*	∼41.5	16.3	95.6
*NTCP*	31.6	4.15	119
*MRP2*	>50	∼51.0	>200
*MRP3*	>50	∼44.6	61.2
*MRP4*	>50	4.26	37.9

There are several questions to be addressed in order to further evaluate the strength of this hypothesis as the solitary mechanistic cause of tolvaptan-induced liver injury. First is the role of compensatory/adaptive mechanisms in terms of transcription/posttranscriptional events that may serve to nullify (or not) the effect of bile acid transporter inhibition (Wagner and Trauner 2005), and the role of disease in determining this response. Second is the prolonged nature of the liver injury that occurs; as outlined in Fig. 4, liver injury continues to progress over a prolonged period even following the withdrawal of the drug. Third is the rapid recurrence upon rechallenge with the drug; whereas a disease threshold explanation could offer insight into the long initial latency to DILI and the reproducibility of a reaction without such a prolonged latency, the toxicokinetic profile of the relevant compounds would need to be reconciled with the rapidity of injury even despite the lower doses used in rechallenge events in patients, e.g. Fig. 4.

### Evidence for a multifaceted mechanism of hepatic insult

Given that neither direct cellular stress mechanisms nor the bile acid hypothesis offers a comprehensive explanation for the observed DILI with tolvaptan, it appears likely that multiple mechanisms are at play in eliciting hepatic insult. Quantitative systems toxicology interfaces such as DILIsym can be used as a malleable tool to integrate multiple parameters from isolated mechanistic toxicology and pharmacokinetic studies, and can ultimately model an expected outcome in terms of predicted toxicity. A detailed overview of DILIsym is not within the remit of this manuscript and a discussion of the model composition and application is provided elsewhere (Watkins 2019, 2020). Briefly, DILIsym is a model to evaluate hepatotoxicity through the integration of pharmacokinetic/exposure data, mechanistic hepatotoxicity data (drug effects on bile acid transporters, mitochondrial respiration, and ROS generation), and application of a simulated patient population that exhibits inherent intraindividual variability. Submodels of DILIsym include physiologically based pharmacokinetics (PBPK), mitochondrial, bile acid, reactive nitrogen/oxygen species, hepatocyte life cycle, and simulated populations. With regards to tolvaptan, DILIsym (Woodhead et al. 2017) was applied with the aim of aiding understanding of how mechanisms captured in DILIsym may have contributed to the observed DILI (Table 5). In vitro data were generated and used to inform the QST model in terms of simple mechanistic parameters of toxicology with respect to tolvaptan, which included:

**Table 5.** Overview of key elements of DILIsym study designs and findings referenced.

Study reference	Key populations of interest in study design (daily dose, compound, and duration details)	Simulated DILI output (ALT >3× normal/Hy’s law) as % of patients	Key finding(s)
(Woodhead et al. 2017)	Standard simulated population (60 mg daily dose of tolvaptan)	(0.4/0.4)	Renally impaired populations more susceptible to DILI than renally sufficient populations Indication of multiple mechanisms capable of driving toxicity
	Standard simulated population (90/30 mg daily dose of tolvaptan)	(7.8/6.6)	
	Renally impaired population (90/30 mg daily of tolvaptan)	(30.6/28.8)	
(Beaudoin et al. 2021)	Standard simulated population (90/30 mg daily of tolvaptan)	(0.4/0.4)	Populations with reduced biliary efflux of tolvaptan and DM-4103 (as expected by potential ADPKD disease-related changes in MRP2 expression) are more susceptible to DILI DM-4103 metabolite is the main driver of differential findings
	Simulated population with 3-fold MRP2-related reduction of efflux of TVP and DM-4103 (90/30 mg daily of tolvaptan)	(3.9/2.1)	
	Simulated population with 3-fold MRP2-related reduction of efflux of TVP and DM-4103 (90/30 mg daily of tolvaptan)	(24.6/14.4)	
(Woodhead et al. 2020)	Standard simulated population (200/100 mg daily of Lixivaptan 12 wk)	(0/0)	No incidence of DILI in simulation Utility of typical population considered representative of both renally sufficient and insufficient population based on limited data to support exposure changes in end-stage kidney disease

- Bile acid transport parameters: Data pertaining to bile acid transporter inhibition data were obtained from the publication outlined above (Slizgi et al. 2016).
- Capacity to perturb mitochondrial function (as measured by mitochondrial respiration assays): data outlined in “Direct toxicological mechanisms” section.
- Reactive oxygen species generation: Experiments evaluating ROS generation within hepG2 cells in vitro (as measured using dihydroethidium) yielded no significant ROS generation in 24-h incubations (tolvaptan 0.01–50 μM, DM-4103 0.01–200 μM, DM-4107 0.01–200 μM) (Woodhead et al. 2017).

These parameters were then contextualized using PBPK modeling informed by pharmacokinetic data from early phase trials and supporting nonclinical tissue distribution data (Shoaf et al. 2007, 2012; Furukawa et al. 2011; Woodhead et al. 2017). Recapitulation of the clinical scenario was modeled (60 mg daily dose for renally sufficient individuals as in hyponatremia, and 90/30 mg split for renally insufficient individuals as in ADPKD). The outputs of simulations reflected findings from the clinical trials: Virtual absence of toxicity in the non-ADPKD scenario, and incidences of ALT elevations and potentially serious DILI in the ADPKD scenario.

Specific mechanisms underlying the simulated DILI were assessed in the model through effectively switching off data inputs (modes of toxicity and parent drug/metabolite contributions). From this, it was concluded that converging multifactorial mechanisms of toxicity were potentially causative of the DILI etiology. Multiple aspects were found to contribute to the observed DILI, including disruption of bile acid homeostasis and mitochondrial respiration (Woodhead et al. 2017). Although conclusions derived from the application of DILIsym software are tangible and have made considerable contributions to the knowledge base, it is important to take into consideration the limitations of software as a model of such a complex biological system (i.e. only as good as the data input). As noted in the study, the software utilized often does not incorporate compensatory mechanisms of liver biochemistry which would protect against the aforementioned insults. In the absence of such mechanisms, it is therefore expected that simulations will overestimate the prevalence of DILI. Indeed, an inflated number of ALT elevations and Hy’s law cases was delivered by the simulations (Woodhead et al. 2017) relative to actual incidence in the TEMPO trials (Watkins et al. 2015). It is not yet clear to what extent these compensatory mechanisms would nullify the effects of bile acid accumulation and mitochondrial dysfunction as causative mechanisms of tolvaptan-induced liver injury.

An in vivo Collaborative Cross study using genetically diverse and inbred mice populations (a total of 45 strains) was also conducted with the aim of identifying genetically driven susceptibility factors/mechanisms of toxicity in relation to tolvaptan (Mosedale et al. 2017b). All of these strains were exposed to a single oral 100 mg/kg dose of tolvaptan, with 3/45 strains identified as susceptible due to observation of ALT elevations on the study. The key findings from this study reinforced support for at least 2 of the aforementioned possible mechanisms. Upon transcriptomic profiling of the liver, it was found that several pathways were significantly altered by tolvaptan, with further value in terms of specificity added by identifying which pathways were exclusively altered in sensitive strains. Firstly, in line with the bile acid mechanisms outlined above, “FXR/RXR activation” and “Bile acid biosynthesis” were identified as key pathways associated with ALT elevations. Secondly, several pathways involved in the immune response: “acute phase response signalling,” “chemokine signalling,” and “CXCR4 response” were associated with tolvaptan treatment across all strains. Notably, differential upregulation in “antigen presenting pathway” genes featured in the most significantly upregulated pathways enriched in susceptible strains relative to nonsusceptible strains. Key proteins highlighted as significant differentially expressed following tolvaptan treatment in this study were secretory leukocyte peptidase inhibitor (slpi) and whey acidic protein 4-disphide-core 12 (Wfdc12), both of which are known to modulate inflammation. The fold change in transcript levels of slpi correlated with tolvaptan exposure and could further differentiate between sensitive and resistant strains, and correlated with ALT elevations (Mosedale et al. 2017b). Slpi serves to inhibit the proinflammatory response (Taggart et al. 2005; Xu et al. 2007), and opposes the action of TGF-B; inhibiting differentiation of regulatory T-cells (Tateosian et al. 2011; Müller et al. 2012). Slpi was therefore identified as a potential link between initial hepatic insult and the stimulation of innate immunological activation which could ultimately initiate and propagate an adaptive immune response.

Taken together, these more holistic studies point toward a multifactorial etiology of tolvaptan-associated DILI, with multiple mechanisms contributing to a cumulative hepatic insult which may drive liver injury itself. Alternatively, these mechanisms may collectively provide the preconditions necessary for the initiation of a deleterious adaptive immune response.

### The role of the adaptive immune system

The common clinical features of tolvaptan-associated liver injury (complex dose-toxicity relationship, long latency of onset, and the rapid recurrence of liver injury upon rechallenge) were considered to indicate possible involvement of the adaptive immune system (Watkins et al. 2015). Observations of cytolytic hepatitis (Watkins et al. 2015) and immune infiltrate upon histological assessment of liver biopsies taken from patients with tolvaptan-associated liver injury lend are also consistent with this notion (Endo et al. 2019).

Dedicated investigative studies were conducted with a view to understand and evaluate a potential role of the adaptive immune system in tolvaptan-associated DILI. These studies identified memory T-lymphocytes responsive to tolvaptan (metabolites) within circulating peripheral blood mononucleated cells sampled from TEMPO 3:4 (NCT00428948) trial participants who exhibited liver injury (Gibson et al. 2020). This provided evidence for the mechanistic involvement of T-cells and supports a role for the adaptive immune response within the observed reactions (Gibson et al. 2020). These T-cells were subsequently characterized and were demonstrated to secrete cytokines and cytolytic effector molecules such as granzyme B upon stimulation with drug (metabolites), indicating a capacity for compound-specific cytolytic activity. Such effector functions are considered to be a critical mechanism by which tissue damage can be elicited in type IV hypersensitivity reactions and reflect similar findings in several studies of immune-mediated IDILI associated with other drugs (Mennicke et al. 2009; Monshi et al. 2013; Wuillemin et al. 2014; Kim et al. 2015; Nattrass et al. 2015). The drug-responsive T-cells residing within DILI patient circulation were found to be predominantly responsive to the hydroxybutyric metabolite DM-4107, indicating that this metabolite may be important for antigenicity. Subsequent in vitro investigations evaluated the intrinsic immunogenicity of tolvaptan, DM-4103, and DM-4107 within healthy donor-derived culture platforms (Hammond et al. 2021a). In these studies, T-cell priming assays were utilized (which are prototypical assays for evaluation of preclinical immunogenicity of small molecular weight drugs, and are used with increasing frequency within immunogenicity risk assessment of biologicals) (Hammond et al. 2021b; Gokemeijer et al. 2023). T-cells responsive to tolvaptan, DM-4103, and DM-4107 were generated in these assays, indicating the antigenicity of these compounds and thus a capacity to elicit de novo adaptive immune responses. In clonal characterization studies, most drug-responsive T-cells responded to DM-4107, and the T-cells tended to exhibit similar functional characteristics to those derived from patients (Hammond et al. 2021a). The importance of these studies is that they clearly demonstrate the potential for inception and elicitation of an adaptive immune response directed against tolvaptan (metabolites), and demonstrate unequivocally that drug-responsive T-lymphocytes were present in DILI patients.

Thus, an enticing proposition that links all of the above concepts is that of initiating steps followed by an adaptive immune attack as the “executioner” mechanism. The mechanisms described above (perturbation of bile acid homeostasis, disruption of mitochondrial respiration, and other direct toxicological mechanisms) could individually or synergistically induce initial hepatic insult. This could then lead to the release of damage-associated molecular patterns and the activation of local innate immune cells, establishing a “danger” environment conducive to the priming of a deleterious adaptive immune response within susceptible individuals.

## The role of ADPKD and other risk factors influencing susceptibility

An unusual feature of tolvaptan-associated liver injury is that it is known to be largely restricted to individuals treated for the indication of ADPKD. Therefore, careful consideration must be given to attributes of these individuals that may influence their susceptibility to DILI. Regarding the proposition that tolvaptan-associated liver injury is the product of an adaptive immune response (hypersensitivity reaction), epidemiological differences in the frequency of drug hypersensitivity reactions are known to occur in general and can be dependent on various patient-related factors. To date, enhanced rates of hypersensitivity have been observed in patients with HIV, e.g. sulfamethoxazole hypersensitivity (Coopman et al. 1993; Phillips and Mallal 2007), and in cystic fibrosis (piperacillin hypersensitivity) (Wills et al. 1998), both likely dependent upon patient immune status. Induction of susceptibility within patient cohorts through medicinally induced immune-regulatory perturbation has also been identified within recent years (Hammond et al. 2022b; Grice et al. 2024).

It is not well-defined at present whether ADPKD patients exhibit greater hypersensitivity rates (at least in the form of idiosyncratic liver injury) than other populations for general pharmaceuticals. As such, a cautionary note regarding the assessment of tolvaptan in terms of general hypersensitivity liabilities comes through its status as an orphan drug for ADPKD. An important consideration is therefore what role the indication of ADPKD plays with regards to the DILI profile of tolvaptan. This could serve 2 purposes: Firstly, for the good of tolvaptan itself; identification of key disease-related parameters that could be assessed/monitored may lead to greater informed management and/or mitigation of patient risk. Secondly, an appreciation of what features of ADPKD influence drug tolerability may contribute to the understanding of desirable attributes in future drug design as the field hopes to populate the armamentarium for ADPKD. Key identified ADPKD aspects that may have a risk-modifying effect in this context are discussed below.

### Drug disposition

The first and most simple consideration with regards to the indication is the dose used. The highest dose prescribed for ADPKD (up to 120 mg daily as 90/30 split dosing) exceeds that for other indications (up to 60 mg daily). Thus, simple exposure intensity may play a role. One could consider the lack of a clear correlation observed between tolvaptan dose and liver injury (Watkins et al. 2015) as contradictory to this idea. Alternatively, the necessity for the concomitant presence of other susceptibility factors may offer an explanation for a complex dose relationship.

ADPKD itself may contribute to a distinct pharmacokinetic profile to that observed in other indications. The most obvious disease-related perturbation of PK characteristics is the progressive renal impairment which occurs in line with disease progression. Low renal function has been demonstrated to result in up to 90% greater exposure (AUC) to tolvaptan (Shoaf et al. 2014) and may have important ramifications for metabolites which are excreted renally, namely DM-4107 (Sorbera et al. 2002). With relevance to the potential immune-mediated mechanism, DM-4107 appears to be the most relevant compound in terms of antigenicity in both patients with DILI and in intrinsic immunogenicity studies (Gibson et al. 2020; Hammond et al. 2021a). Poor renal clearance of this metabolite may therefore have an important role in surmounting a theoretical antigenic threshold required for T-cell activation. In a general sense, renal impairment contributing to susceptibility of patients to hypersensitivity reactions is not unprecedented. Indeed, reduced renal function has been demonstrated to play an important role within allopurinol hypersensitivity reactions, with reduced eGFR contributing a consistent, graded odds ratio for reactions across expression status' of the HLA-B*58:01 risk allele (Ng et al. 2016). Interestingly, one of the only cases of tolvaptan-associated liver injury reported in non-ADPKD indications was in an individual with stage III chronic kidney disease (Khan et al. 2019). Alternatively, it is also conceivable that patients with diminished renal capability could experience anomalously high concentrations of otherwise irrelevant metabolites that have not been well-studied to date, though identification and quantitation of such species remain poorly resolved.

The role of renal impairment in influencing the extent of exposure and thus downstream hepatic insult has been modeled using DILIsym (Woodhead et al. 2017). Comparison of simulations conducted on renally sufficient and impaired populations yielded a considerable increase (>3- and >4-fold increases for simulated ALT 3× ULN and Hy’s law cases respectively) in the incidence of predicted liver injury (Woodhead et al. 2017) (Table 5). One key message from this simulation was that renal deficiency could have a detrimental on the toxicological profile of tolvaptan. In later simulations (which are discussed in greater detail below) a renally sufficient population was used, but with anticipated ADPKD conferred changes in bile acid transporter function, i.e. lower MRP2 activity (Beaudoin et al. 2021) (Table 5). It would be interesting (and possibly a useful exercise in further understanding of the model) to evaluate just what level of predicted liver injury would arise from dual MRP2 and renal effects in the 90/30 regimen across the QSP model.

In addition to renal-mediated PK effects, greater hepatic exposure of tolvaptan, DM-4103, and DM-4107 has been noted in isolated perfused rodent livers of PCK rats relative to WT, suggesting that disease-specific accumulation of these compounds occurs in the liver (Beaudoin et al. 2019). On the subject of hepatic exposure and metabolism, DM-4103 and DM-4107 are derived from the parent drug via similar metabolic pathways and so would be likely to co-localize in a manner that potentially promotes synergistic accumulation and toxic sequela.

### Mechanism-related factors

Other aspects of ADPKD disease may have sensitivity-modifying effects on the toxicological mechanisms identified. As highlighted above, there is a potential disease interaction with respect to bile acid transport, namely the alterations in bile acid transporters and deviation of bile acid homeostatic conditions in ADPKD populations relative to other patient cohorts. The relevance of disease-dependent changes in MRP2 expression have been modeled following extrapolation of in vivo and in vitro model-based findings via quantitative systems toxicology and do appear to indicate an important role for dysfunctional biliary efflux seen in ADPKD patients (Beaudoin et al. 2021) (Table 5).

Another observation is that a common extrarenal manifestations/associations of ADPKD is hepatic cyst formation (Reynolds et al. 2000), described as polycystic liver disease (PLD). PLD affects between 22% and 95% of patients with ADPKD, increasing in line with advancing age (Bae et al. 2006). Notably, a greater prevalence of severe PLD is detected in females (Cnossen and Drenth 2014; Hogan et al. 2015), which could be of relevance to the higher reported DILI rate in female subjects. Moreover, in patients for which T-cell responses were evaluated, all 6 patients subject to liver cyst investigation were confirmed to have a positive status (Gibson et al. 2020). An important point pertaining to liver cysts is that the progression of PLD is unaffected by the administration of tolvaptan (due to the absence of V_2_R in the liver) (Torres and Harris 2009). Progressive hepatic architecture disruption is therefore probable even in the setting of efficacious tolvaptan treatment in the kidney, and could potentially contribute to hepatocyte stress and localized danger signaling. The progression, and complications of these cysts could also play a role in the variable, often long latency periods of up to 18 months exhibited by patients presenting with DILI (Watkins et al. 2015). To date, however, no formal link between hepatic cyst presence and tolvaptan-associated liver injury has been established (Alpers et al. 2023).

A heightened inflammatory state has been reported in ADPKD individuals, with key inflammatory mediators such as monocyte chemoattractant protein-1 and tumor necrosis factor-α identified to date (Ta et al. 2013). Further support for an inflammatory component in ADPKD patients is provided by the aforementioned upregulation of slpi in susceptible mice strains during tolvaptan treatment (Mosedale et al. 2017b), and the significantly greater presence of inflammasome signaling expressed in ADPKD cells (de Almeida et al. 2016). In a study evaluating the proteomic contents of urinary extracellular vesicles, it was found that tolvaptan treatment of patients led to significant upregulation of several (a total of 25) proteins, 4 of which were immunoreactive (IGHG1-3 and IGKC) (Pocsfalvi et al. 2015). An important question to address is whether this is mirrored by hepatic extracellular vesicles/exosomes, as indicated by murine studies (Mosedale et al. 2017b), and what pathogenic role (if any), disease-related inflammation may play.

## A working hypothesis

**Fig. 5.**
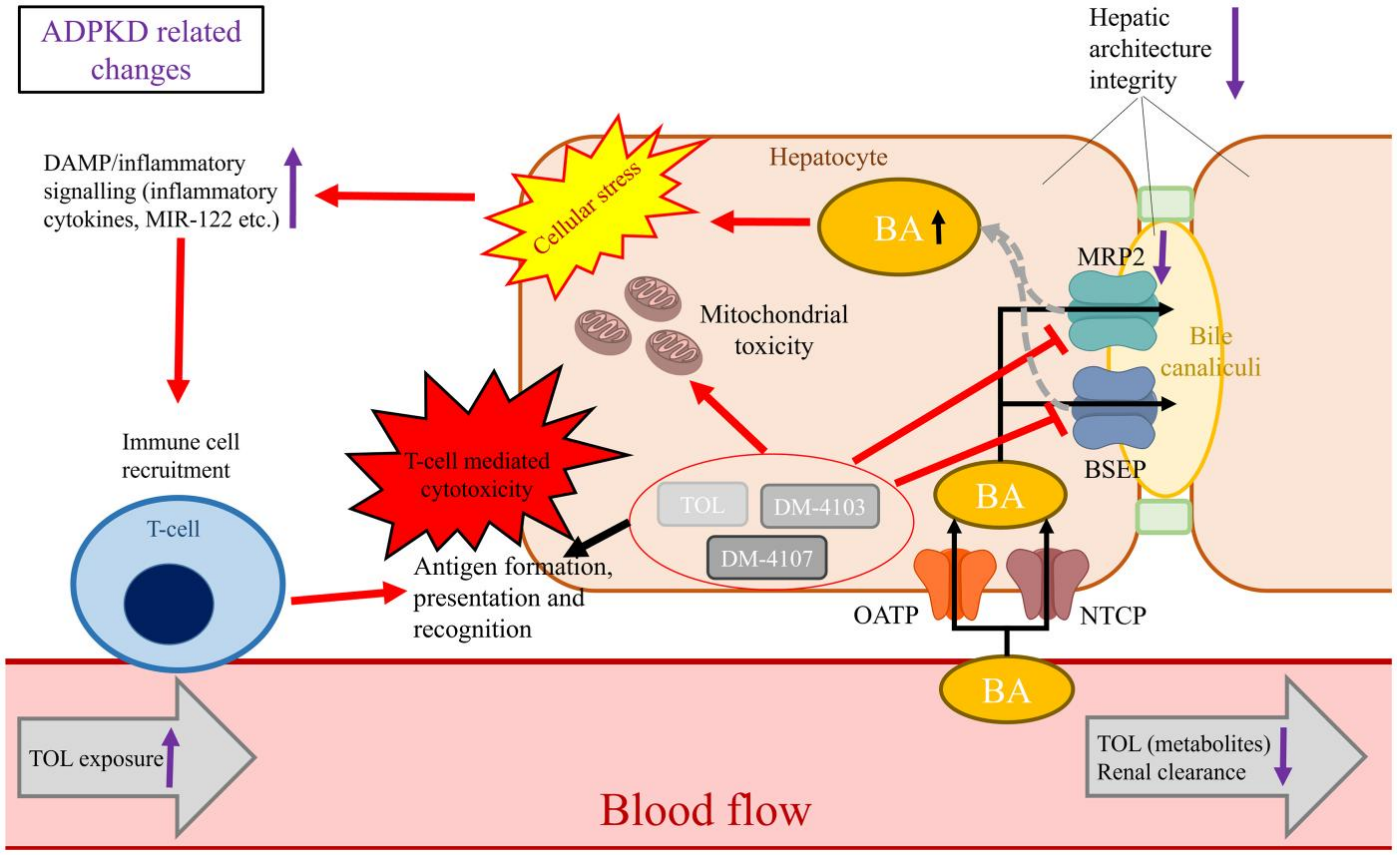
Working hypothesis for tolvaptan-induced liver injury. A constellation of mechanistic toxicity pathways (bile acid accumulation, hepatocellular stress, mitochondrial toxicity), exacerbated by ADPKD-related changes (exposure, transporter expression, inflammatory milieu, and hepatic architecture) leads to the possible elicitation of an immune-mediated DILI reaction in susceptible individuals. Dark arrows adjacent to elements indicate ADPKD-related changes in an individual’s physiology that may contribute to their overall susceptibility. Dashed arrows indicate the redirection of bile acids away from biliary efflux.

Incorporating all of the above elements; direct toxicological mechanisms, potential ADPKD-conferred susceptibility factors, and the involvement of the adaptive immune system, may present a useful working hypothesis for the exclusivity of drug-induced liver injury within ADPKD patients. Greater exposure to tolvaptan and metabolites and/or increased susceptibility to the defined toxicological mechanisms may serve as initiating factors that drive an adaptive immune response directed against drug-related antigens in susceptible individuals. Furthermore, an augmented epitope density may be generated in hepatic tissues due to alterations in drug disposition (Fig. 5).

## Future clinical and developmental challenges

Investigation of the mechanistic basis for tolvaptan-associated DILI is an important pursuit. Ultimately, evaluation of each drug with idiosyncratic DILI liabilities for potential mechanisms at play is important so that: (i) understanding is gained with respect to the continued development and clinical management of the drug and (ii) the (often complex) information can be relayed back to discovery/development for accurate utility in preclinical assessment of such liabilities in drug discovery/development.

With respect to tolvaptan itself, the tolerability profile of this drug is considered favorable despite the rare incidence of serious hepatic reactions. Furthermore, it is likely that the REMs strategy has played a role in refining the safety profile of the drug further through early identification and management of liver injury. It is probable that cases of DILI have been avoided through the detection of clinical chemistry signals. Looking forward, there are a number of questions that should be addressed with the continued usage of tolvaptan.

One key aspect is whether a single or multiple critical susceptibility factors to IDILI are identifiable in patients, and if so, whether those factors could enable patient stratification in a manner which mitigates risk. Interesting avenues of exploration with regard to this include pharmacogenetic/phenotypic-based precision medicine approaches. A key piece of evidence which has proven useful for several T-cell-mediated reactions to drugs is the association of such reactions with specific alleles in human leukocyte antigens (HLAs). HLA alleles associations with such reactions have been defined with flucloxacillin (Daly et al. 2009), amoxicillin-clavulanate (Lucena et al. 2011), and potentially ximelagatran (Kindmark et al. 2008), among others. However, despite HLA genotyping being conducted on a cohort of tolvaptan associated liver injury patients, no clear pattern or candidate allele has emerged thus far. To date, the cohort evaluated for these genetic factors is relatively small, and as such, the power of any analysis is therefore limited; clear relationships of this nature are rarely defined until larger cohorts of patients are evaluated. Conversely, there are numerous drugs which do cause T-cell mediated reactions (including DILI) which are not associated with an HLA risk allele. Vigilance must be exercised to detect emerging patterns as clinical experience accrues with tolvaptan, as HLA associations and prescreening methods have proven effective in improving safety outcomes of drugs which cause hypersensitivity reactions (Alfirevic and Pirmohamed 2017). Further lines of investigation for the associative study could include the role of hepatic cysts and how ADPKD-mediated abnormalities in hepatic architecture may be linked to DILI, as well as evaluation of functional phenotypes such as renal status, bile acid transporter status, concomitant medications, and metabolizing phenotype. Down the line, it may be prudent to evaluate whether some of the mechanistic biomarkers evaluated in the preclinical studies overviewed herein, e.g. miR-122 (Mosedale et al. 2017a) exhibit greater sensitivity for early liver injury detection.

An immediate clinical issue is the management of ADPKD in tolvaptan-intolerant individuals following the incidence of IDILI. With tolvaptan considered an orphan drug at the time of writing, the current outlook is bleak. These individuals do not have an alternative approved therapeutic option. Therefore, loss of the ability to modify disease course with tolvaptan due to contraindication may mean that an individual will experience a natural disease course to end-stage renal disease. Rechallenge with tolvaptan following DILI has poor outcomes (even comparative to experience with other idiosyncratic liver injury-causing drugs); over half of individuals experience a recrudescence of liver injury with re-exposure (Hunt et al. 2017). This poor rechallenge rate has been reproduced with multiple cohorts of patients (Watkins et al. 2015; Alpers et al. 2023), and the secondary reaction also has the potential to be more severe (dose relative) than the original reaction as seen in Fig. 4. Looking towards pharmaceutical development in the field, the most logical solution for these individuals is referral to a compound with a similar mechanism of action. In line with this, investigations have been launched for the therapeutic use of alternative vaptans in ADPKD. The “front runner” of these potential options was lixivaptan for several years. In the development of lixivaptan, quantitative systems toxicology modeling (DILIsym) was employed in the prediction of toxicities pertaining to lixivaptan for ADPKD (Woodhead et al. 2020). As with the modeling of DILI associated with tolvaptan (Woodhead et al. 2017; Beaudoin et al. 2021), the model permitted the interpretation of multiple direct toxicological mechanisms including bile acid transporter inhibition and oxidative stress. The outcomes of this study were very encouraging, as a superior simulated toxicity profile for lixivaptan was observed (in fact the absence of simulated DILI was seen) (Woodhead et al. 2020) (Table 5). Clinical development followed, with lixivaptan evaluated for the treatment of ADPKD circa 2019 in multiple clinical trials (NCT03487913, NCT04064346, and NCT04152837). Importantly, the latter of these, the ALERT trial (NCT04152837) was directly informative to the question of whether safe referral could be possible, as it specifically enrolled tolvaptan-intolerant individuals. Unfortunately, safety signals in the form of ALT/AST elevations were identified in this ALERT trial, and likely played a key role in the decision of the sponsor (Centessa) to discontinue lixivaptan’s development in this area, terminating the phase III trials in ADPKD at early stages (Centessa Pharmaceuticals 2022). The profile of the liver injury encountered with lixivaptan is not publicly available at present, but appears to have only been observed in a patient previously treated (and exhibiting liver chemistry signals) with tolvaptan; the ALT elevations were seen only in the ALERT and not the ACTION trial. A key question that arises is whether these findings would have been observed in individuals without previous intolerance to tolvaptan, and if so, what makes tolvaptan-intolerant individuals uniquely sensitive to tolvaptan and lixivaptan? Further to this is why clinical findings were in contrast to in silico predictions.

A possible explanation for a reaction to lixivaptan in an individual with previous tolvaptan intolerance could be immunological cross-reactivity between tolvaptan and lixivaptan (or respective metabolites). Simply described, in tolvaptan hypersensitive individuals, memory T-cells responsive to tolvaptan may also respond to lixivaptan if the relevant drug-associated antigens are structurally similar enough. Should this be the case, these individuals would have secondary intolerance to lixivaptan, despite never being exposed directly to lixivaptan itself. Importantly, this could occur regardless of the capacity of lixivaptan to generate a de novo T-cell response. Hypersensitivity literature frequently describes such structurally driven collateral sensitization. A short report attempted to address this question by evaluating T-cell cross-reactivity across parent Vaptan drugs (Hammond et al. 2023). Precedence for cross-reactivity within the Vaptan class was indeed seen in this study. Vigorous responses were seen with T-cells primed to tolvaptan when they were exposed to the structurally related drug mozavaptan. No cross-reactive responses were observed for lixivaptan in this study. However, limitations of this study included a lack of comprehensive coverage of metabolite cross-reactivity (as the in vitro systems were not metabolically competent), and a limited number of donors from which T-cells were derived (Hammond et al. 2023). Thus, the possibility of donor-dependence in cross-reactive status, or cross-reactivity to a lixivaptan metabolite could not be ruled out. On this line, the actual prevalence of ALT/AST elevations with lixivaptan secondary to tolvaptan (NCT04152837 results) does appear to be significantly lower than the observed rate of rechallenge reactions with tolvaptan. Therefore, if immunological cross-reactivity was responsible for the observed liver injury in those trials, it is possible that it is only applicable to a restricted subpopulation of tolvaptan-intolerant individuals. To summarize, the immunological cross-sensitivity explanation offers a plausible explanation for the restriction of liver injury to only those previously intolerant to tolvaptan. It also accounts for the failure to accurately predict such issues in QST models due to the absence of adaptive immune representation in such models. Although precedence for such cross-reactivity across the vaptan class has been experimentally validated, no data has been generated to date which directly supports tolvaptan-lixivaptan (or metabolites thereof) immunological cross-reactivity. Nor has the clinical presentation of lixivaptan-elicited liver chemistry signals been reconciled with a rechallenge-like immune response to date. With respect to novel therapeutics that are to be used secondary to the identification of tolvaptan intolerance, whether testing T-cells primed to tolvaptan for prospective cross-reactivity is feasible and/or the extent to which findings could add confidence to a development program is ill-defined. Alternatively, it is conceivable that additional mechanistic or patient susceptibility factors that have not been defined to date drive the observed lixivaptan-associated liver signals. In any case, data relating to lixivaptan-associated liver safety signals and causal mechanisms thereof are scant.

## Conclusions

To conclude, tolvaptan is an important drug, and is currently the solitary agent approved for ADPKD which is known to possess disease-modifying efficacy for this indication. It is safe and efficacious in the majority of treated patients but is known to cause idiosyncratic liver injury in a small fraction of individuals. The pathomechanistic aspects behind this liver injury have not been conclusively resolved, but are most likely multifaceted and exhibit an indication-specific bias. Through the fruits of multidisciplinary efforts in mechanistic investigation, we present herein a plausible working hypothesis for the observed liver injury. It is desired that this hypothesis be challenged, as the closer to the truth we move, the better placed we are to understand and ultimately mitigate such liver injury. Looking to the future, tolvaptan is here to stay as a therapeutic for ADPKD, and as understanding moves forward, it is hoped that additional patient susceptibility factors are identified and acted upon, thus aiding the further refinement of the safety profile of this important therapeutic.
